# Prediction of axial elongation in adults with high myopia: the Wenzhou High Myopia Cohort Study

**DOI:** 10.1186/s40662-026-00496-y

**Published:** 2026-06-22

**Authors:** Yuan Wang, Xue Rui, Yue Sun, Yan Li, Shihao Ding, Zhilu Zhang, Jie Lu, Meixin Wu, Jia Qu, Xiangtian Zhou, Hao Wu

**Affiliations:** 1 Oujiang Laboratory (Zhejiang Lab for Regenerative Medicine, Vision, and Brain Health), State Key Laboratory of Eye Health, National Clinical Research Center for Ocular Diseases, Eye Hospital, Wenzhou Medical University, Wenzhou, Zhejiang China; 2 State Key Laboratory of Eye Health, Department of Ophthalmology, Shanghai Key Laboratory of Orbital Diseases and Ocular Oncology, Ninth People’s Hospital, Shanghai Jiao Tong University School of Medicine, Shanghai, China

**Keywords:** High myopia, Axial elongation, Predictors, Choroidal thickness, Age

## Abstract

**Background:**

Identifying individuals at high risk of axial elongation is crucial for managing adults with high myopia, as further elongation substantially increases the risk of vision-threatening complications. This study aimed to identify the optimal predictors for axial elongation in this population.

**Methods:**

This prospective cohort study enrolled 1025 eyes from 532 adults with high myopia (18–60 years). We evaluated the predictive performance of nine candidate variables for rapid axial elongation, defined as a 2-year axial elongation of at least 0.10 mm. Predictors were selected using bidirectional stepwise regression. Model discrimination was assessed using the area under the receiver operating characteristic curve (AUC), and the incremental value of the new model was quantified using integrated discrimination improvement (IDI).

**Results:**

The mean annual axial elongation rate was 0.020 ± 0.051 mm/year, and 24.78% of eyes developed rapid axial elongation. Stepwise regression constructed a full model (AUC = 0.811) including six predictors—age, sex, axial length, best-corrected visual acuity, choroidal thickness (ChT), and vascularity index. ChT showed the highest individual discriminative ability (AUC = 0.728), followed by age (AUC = 0.666). Removing age or ChT from the full model reduced the AUCs to 0.735 and 0.753, respectively (both *P* < 0.001), with IDIs of  − 0.116 and − 0.095 (both *P* < 0.001). In contrast, excluding sex only resulted in an IDI of − 0.008 (*P* = 0.032). Simplified models combining age and ChT, with or without sex, showed discrimination comparable to that of the full model, with only modest reductions in predictive performance (IDIs: −  0.028 and − 0.033, respectively; both *P* < 0.05). In the bivariate model, the predictive cut-off of ChT decreased progressively with age, ranging from 187 µm at age 20 to 29 µm at age 60. The results remained consistent in sensitivity analyses using alternative axial elongation definitions (0.05 mm and 0.15 mm). Nonlinear relationship analyses revealed that thinner choroids were associated with faster axial elongation rate only below an inflection point.

**Conclusions:**

ChT and age are the optimal predictors for axial elongation in adults with high myopia. These findings establish their dominant role in personalized management and support further investigation into choroidal architecture to guide proactive, choroid-centered strategies.

*Trial Registration*: This study was registered with the Chinese Clinical Trial Registry (ChiCTR2100047424).

**Supplementary Information:**

The online version contains supplementary material available at 10.1186/s40662-026-00496-y.

## Background

Myopia progression is predominantly driven by axial elongation, which follows a characteristic age-dependent trajectory. Axial growth is rapid during childhood, gradually slows during adolescence, and typically plateaus by adulthood, with axial length (AL) stabilizing in most individuals [1]. However, axial elongation persists into adulthood in approximately 11.7% to 40% of individuals with high myopia [2–5], substantially increasing the risk of irreversible vision-threatening complications, such as myopic maculopathy and retinal detachment [6]. Therefore, identifying adults with high myopia who are at high risk of progressive axial elongation is essential.

Recently, increasing attention has been directed toward myopia progression in adults [7, 8]. A number of potential risk factors for progressive axial elongation have been reported, including age [3, 4, 6, 9], sex [3, 4, 10, 11], baseline AL [3, 4, 9, 12], baseline spherical equivalent refraction (SER) [9], visual acuity [3, 6], intraocular pressure (IOP) [4, 13], choroidal thickness (ChT) [14], and the presence and type of myopic macular degeneration (MMD) [3, 6, 10, 11, 14]. These findings have improved our understanding of myopia progression; however, they offer limited direct clinical applicability. Specifically, their actual predictive performance for future axial elongation remains unquantified and poorly validated. This represents a key translational gap: without robust, evidence-based tools to stratify an individual’s progression risk, clinicians have limited ability to implement truly personalized monitoring and targeted interventions.

To address this gap, we conducted a 2-year prospective cohort study integrating multimodal indicators, including baseline ocular parameters and optical coherence tomography (OCT) derived imaging features, to identify key predictors of axial elongation in adults with high myopia. Moving beyond mere identification of risk factors to the validation of their predictive value, this study seeks to clarify which variables possess meaningful clinical utility. Ultimately, our goal is to inform the development of practical, evidence-based risk-stratification frameworks and to promote more individualized approaches to managing axial elongation in adult myopia.

## Methods

### Study population

The Wenzhou High Myopia Cohort Study is an ongoing prospective cohort study conducted at the National Clinical Research Center for Ocular Diseases in the Eye Hospital of Wenzhou Medical University. Participants with high myopia, defined as SER ≤  − 6.00 D (post-cycloplegic autorefraction) or AL ≥ 26.5 mm, have been recruited since November 2021 and followed annually. The methodology has been described previously [15]; this report analyzes data from enrollment to August 2025. In brief, adults with high myopia aged 18 to 60 years at baseline, without secondary myopia, a history of ocular surgery, or severe systemic medical conditions were enrolled. Eyes with available data on AL at baseline and the second-year follow-up visit, and analyzable OCT images at baseline were included in the current analysis. Eyes were excluded if they underwent surgery or received specific treatments for myopia (i.e., corneal laser surgery for myopia) during follow-up. Written informed consent was obtained from all participants. The study adhered to the principles of the Declaration of Helsinki, was approved by the institutional ethics committee of the Eye Hospital of Wenzhou Medical University (2020-199-K-181-07), and was registered with the Chinese Clinical Trial Registry (ChiCTR2100047424). It also followed the Strengthening the Reporting of Observational Studies in Epidemiology reporting guideline.

### Demographics and ophthalmic evaluations

Participants’ demographics and other relevant parameters were assessed at baseline, including age, sex, height, weight, duration of myopia, family history of myopia, and blood pressure. Ophthalmic examinations comprised: slit lamp-based biomicroscopic examination of the anterior segment; IOP using the noncontact tonometer (TX-20; Canon, Tokyo, Japan); biometry for AL (IOL Master 700, Carl Zeiss Meditec, Jena, Germany); best-corrected visual acuity (BCVA) using a logMAR E visual acuity chart; and posterior segment OCT and OCT angiography imaging (VG200D, SVision Imaging, Henan, China). Fundus photography was performed with a digital retinal camera (Zeiss Visucam 224, Germany) after cycloplegia with tropicamide 0.5% and phenylephrine 0.5%. Next, refractive status was measured with an autorefractor (ARK-510A, NIDEK, Japan); the mean of three readings was recorded as the final measurement. All examinations were performed in the afternoon to minimize potential diurnal fluctuation.

### Choroidal imaging and quantification

Choroidal images were obtained using swept-source OCT containing a swept-source laser with a central wavelength of approximately 1050 nm at a scan rate of 200,000 A-scans per second. Choroidal imaging was performed using two radial scan lines oriented at 90° apart, covering a 12-mm diameter circular area centered on the fovea (Additional File 1: Figure S1a), with transverse and axial resolutions of 13 μm and 5 μm, respectively. Each radial line scan included 1024 A-scans and was averaged by 64 B-scans. Automatic eye tracking was enabled during image acquisition, and the follow-up function was applied for all participants at each follow-up visit to ensure scan location consistency.

Image magnification was adjusted using Bennett’s formula [16], based on the AL of each eye. The OCT structural images from horizontal meridians were automatically segmented using a previously established deep learning model that has shown high accuracy with a Dice coefficient of 0.924 [15]. The results of choroidal boundary segmentation were checked twice and manually corrected for segmentation errors and images with severe parapapillary atrophy. Subsequently, ChT was measured as the axial distance between the posterior border of the retinal pigment epithelium and the anterior surface of the chorioscleral interface. Within the choroid, the luminal area (LA) and stromal area (SA) were differentiated through image binarization using the Niblack auto-local threshold algorithm, which calculates a unique threshold for each pixel based on the mean and standard deviation of its local neighborhood. In the resulting binary images (Additional File 1 Figure S1d), the LA was represented by white pixels (vascular spaces), while the SA was represented by black pixels (interstitial connective tissue). The choroidal vascular index (CVI) was calculated as LA/(LA + SA), representing the ratio of LA to the total choroidal area.

### Outcome definition and candidate variables

The primary outcome was rapid axial elongation, defined as a 2-year axial elongation of at least 0.10 mm. Sensitivity analyses were also performed using alternative cut-offs of 0.05 mm and 0.15 mm. Based on an extensive literature review and clinical expertise [3, 4, 6, 9–14], nine candidate predictors were prespecified. These included demographic characteristics (age and sex) and baseline ocular parameters: AL, SER, BCVA, IOP, ChT, CVI, and MMD. The myopic maculopathy was categorized based on the META-PM classification system.

### Statistical analysis

Statistical analyses were performed using R software (V.4.5.0, https://www.r-project.org). A total of 1025 eyes from 532 participants were included in the statistical analysis. Data normality was assessed using the Shapiro–Wilk test. Continuous variables are presented as mean ± standard deviation (SD), and categorical variables as numbers (percentages). Baseline characteristics were compared between eyes with 2-year axial elongation < 0.1 mm and those ≥ 0.1 mm using generalized estimating equations (GEE) with an exchangeable correlation structure to account for inter-eye correlation.

Predictor selection was performed using GEE with an exchangeable correlation structure, with all continuous variables standardized as Z scores, and bidirectional stepwise selection guided by the quasi-likelihood under the independence model criterion. Subsequently, prediction models for axial elongation were developed using a dataset comprising 532 eyes from 532 participants. Multicollinearity was assessed using variance inflation factors; values < 5 indicated acceptable collinearity. The relative contribution of each predictor was assessed using the Wald Z statistics. Simplified models were constructed by sequentially removing one predictor at a time, each differing from the full model by excluding a single variable, and were compared with the full model. Model discrimination was assessed using the area under the receiver operating characteristic curve (AUC), with differences between models evaluated using the DeLong test. The contribution of specific variables to the model’s predictive probability was quantified using integrated discrimination improvement (IDI). By comparing the full model with the simplified versions after stepwise variable elimination, we evaluated the impact of each removed variable on reclassification performance. Internal validation was conducted for the simplified and full models using 1000 bootstrap resamples to estimate the optimism-corrected AUC. Model calibration was evaluated using calibration plots.

Locally weighted scatterplot smoothing (LOWESS) plots were generated to visualize the relationship between baseline ChT or age and annual axial elongation rate. The association was further modeled using generalized additive mixed models (GAMMs) with subject-level random intercepts (using the mgcv package in R; https://cran.r-project.org/package=mgcv) to evaluate potential nonlinear relationships.

## Results

### General characteristics of the study population

**Fig. 1:**
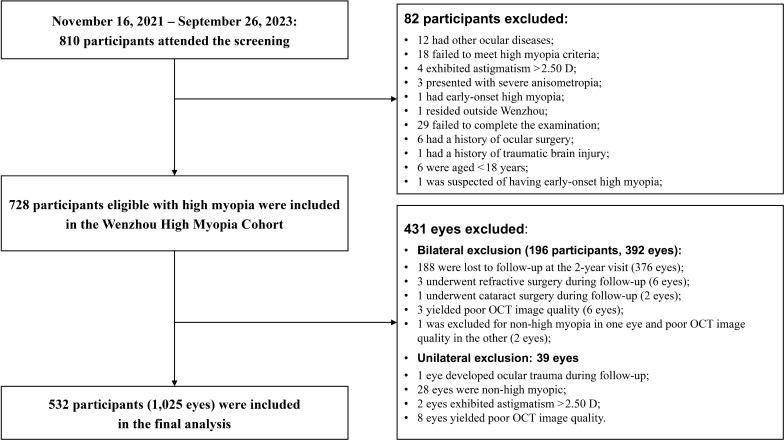
Flowchart of the data acquisition process and data analysis

Of the 810 patients who attended the screening, data from 532 individuals were included in the final analysis, comprising 511 right eyes and 514 left eyes (Fig. 1). The mean age of the participants was 36.14 ± 9.83 years, with 382 females (71.8%) and 150 males (28.2%). Across all participants, the mean AL was 27.02 ± 1.15 mm, the mean SER was − 8.46 ± 2.03 D, and the mean annual axial elongation rate was 0.020 ± 0.051 mm/year over the 2-year follow-up period. Among the included eyes, 594 (57.95%) exhibited an axial elongation of < 0.05 mm, 177 (17.27%) of 0.05 to < 0.1 mm, 111 (10.83%) of 0.1 to < 0.15 mm, and 143 (13.95%) of ≥ 0.15 mm. Table 1 summarizes the demographic and biometric characteristics of participants’ eyes at the baseline examination.

**Table 1:** Baseline characteristics of participants

Characteristics	All participants	Axial elongation < 0.1 mm	Axial elongation ≥ 0.1 mm	*P* value^a^
No. of participants	532	433	163	—
Age (year)	36.14 ± 9.83	37.27 ± 9.63	33.42 ± 10.15	** < 0.001**
Sex, No. (%)				**0.003**
Female	382 (71.80)	301 (69.52)	131 (80.37)	
Male	150 (28.20)	132 (30.48)	32 (19.63)	
No. of eyes	1025	771	254	/
AL (mm)	27.02 ± 1.15	26.89 ± 1.05	27.42 ± 1.34	0.209
SER (D)	− 8.46 ± 2.03	− 8.21 ± 1.79	− 9.22 ± 2.47	**0.037**
IOP (mmHg)	14.57 ± 2.79	14.60 ± 2.74	14.49 ± 2.94	0.682
BCVA (logMAR)	− 0.03 ± 0.06	− 0.03 ± 0.06	− 0.02 ± 0.06	**0.022**
ChT (μm)	155.91 ± 57.36	166.53 ± 57.46	123.70 ± 43.41	** < 0.001**
CVI (%)	61.92 ± 2.90	61.91 ± 2.63	61.95 ± 3.63	0.624
Category of myopic maculopathy, No. (%)				**0.003**
C0/1	839 (81.85)	654 (84.82)	185 (72.83)	
C2/3/4 or plus lesions	186 (18.15)	117 (15.18)	69 (27.17)	

^a^ *P* values were adjusted for inter-eye correlation

*P* values in bold indicate statistical significance

*AL =* axial length; *BCVA* = best-corrected visual acuity; *ChT* = choroidal thickness; *CVI* = choroidal vascularity index; *IOP* = intraocular pressure; *SER* = spherical equivalent refraction

### Screening and performance evaluation of predictive factors for rapid axial elongation

The discriminative performance of individual predictors was assessed separately (Additional File 1 Figure S2). Among the nine variables, ChT showed the highest discriminative ability (AUC = 0.728; 95% confidence interval [CI]: 0.678 to 0.778), with a cut-off value of 138 µm determined by the maximum Youden index. Age (AUC = 0.666; 95% CI: 0.610 to 0.722), SER (AUC = 0.648; 95% CI: 0.592 to 0.704), and AL (AUC = 0.630; 95% CI: 0.573 to 0.688) exhibited modest discriminative ability for predicting rapid axial elongation. Sensitivity analyses using alternative axial elongation cut-offs (0.05 mm and 0.15 mm) yielded consistent results, supporting the robustness of these findings.

Following variable screening using stepwise regression, six of nine candidate predictors—age, sex, AL, BCVA, ChT, and CVI—were retained as core variables in the final multivariable model (Table 2), whereas SER, IOP, and MMD were excluded. Among the retained predictors, ChT and age showed the greatest relative importance within the multivariable model, as indicated by the highest Wald Z statistics (6.28 and 7.08) (Additional File 1 Figure S3). This result remained consistent in the sensitivity analyses using alternative axial elongation definitions (0.05 mm and 0.15 mm) (Additional File 1 Figure S3 and Table 2). Notably, only age, sex, ChT, and BCVA consistently appeared in each model with different cut-offs.

**Table 2:** Multivariable logistic analysis of predictors for rapid axial elongation (≥ 0.1 mm, ≥ 0.05 mm, and ≥ 0.15 mm)

Variable	Model 1 (≥ 0.1 mm)		Model 2 (≥ 0.05 mm)		Model 3 (≥ 0.15 mm)	
	OR (95% CI)	*P* value	OR (95% CI)	*P* value	OR (95% CI)	*P* value
Age	0.34 (0.25, 0.46)	** < 0.001**	0.58 (0.47, 0.71)	** < 0.001**	0.31 (0.21, 0.45)	** < 0.001**
Sex						
Female	Ref		Ref		Ref	
Male	0.59 (0.33, 1.03)	0.071	0.52 (0.33, 0.80)	**0.003**	0.41 (0.19, 0.83)	**0.017**
AL	1.35 (1.03, 1.76)	**0.028**	NA	NA	1.63 (1.20, 2.24)	**0.002**
SER	NA	NA	0.81 (0.65, 1.00)	0.057	NA	NA
BCVA	1.20 (0.95, 1.51)	0.121	1.12 (0.92, 1.37)	0.277	1.15 (0.87, 1.50)	0.305
ChT	0.29 (0.20, 0.42)	** < 0.001**	0.45 (0.35, 0.58)	** < 0.001**	0.40 (0.26, 0.61)	** < 0.001**
CVI	0.74 (0.58, 0.94)	**0.013**	NA	NA	0.81 (0.62, 1.06)	0.123
IOP	NA	NA	0.94 (0.78, 1.14)	0.545	0.98 (0.76, 1.28)	0.907
Myopic maculopathy	NA	NA	NA	NA	NA	NA

*P* values in bold indicate statistical significance

*AL* = axial length; *BCVA* = best-corrected visual acuity; *ChT* = choroidal thickness; *CI* = confidence interval; *CVI* = choroidal vascularity index; *IOP* = intraocular pressure; *NA* = not available; *SER* = spherical equivalent refraction; *OR* = odds ratio

Then the performance of the full model (AUC = 0.811, 95% CI: 0.769 to 0.852) and that of other models after separate exclusion of age, ChT, sex, AL, CVI, and BCVA were assessed (Table 3). The results indicated that omitting either age or ChT significantly impaired model performance. Specifically, excluding age reduced the AUC to 0.735 (95% CI: 0.685 to 0.786; DeLong test, *P* < 0.001; IDI =  − 0.116, 95% CI:  − 0.149 to − 0.083; *P* < 0.001), while removing ChT yielded an AUC of 0.753 (95% CI: 0.705 to 0.801; DeLong test, *P* < 0.001; IDI =  − 0.095, 95% CI: − 0.125 to − 0.065; *P* < 0.001). Conversely, excluding sex alone yielded an IDI of − 0.008 (*P* = 0.032), whereas excluding the remaining variables (AL, CVI, and BCVA) induced negligible changes that were not statistically significant.

**Table 3:** Discriminative performance and incremental discrimination of models with sequential predictor exclusion

Variable	AUC (95% CI)	*P* value ^a^	IDI	*P* value
** ≥ 0.1 mm**^**b**^				
Full model 1	0.811(0.769, 0.852)	Ref	Ref	
Full − Age	0.735 (0.685,0.786)	** < 0.001**	− 0.116 (− 0.149, − 0.083)	** < 0.001**
Full − ChT	0.753 (0.705, 0.801)	** < 0.001**	− 0.095 (− 0.125, − 0.065)	** < 0.001**
Full − Sex	0.807 (0.765, 0.849)	0.393	− 0.008 (− 0.016, − 0.001)	**0.032**
Full − AL	0.805 (0.762, 0.847)	0.260	− 0.010 (− 0.020, 0.001)	0.085
Full − CVI	0.808 (0.765, 0.850)	0.650	− 0.011 (− 0.024, 0.003)	0.116
Full − BCVA	0.810 (0.768, 0.852)	0.937	− 0.004 (− 0.011, 0.002)	0.201
Age + ChT + Sex	0.794 (0.750, 0.838)	0.084	− 0.028 (− 0.048, − 0.008)	**0.007**
Age + ChT	0.793 (0.749, 0.836)	0.067	− 0.033 (− 0.054, − 0.012)	**0.002**
** ≥ 0.05 mm**^**b**^				
Full model 2	0.742 (0.700, 0.785)	Ref	Ref	
Full − ChT	0.677 (0.631,0.723)	** < 0.001**	− 0.075 (− 0.097, − 0.053)	** < 0.001**
Full − Age	0.708 (0.663, 0.752)	**0.014**	− 0.047 (− 0.065, − 0.029)	** < 0.001**
Full − Sex	0.731 (0.687, 0.774)	0.117	− 0.015 (− 0.025, − 0.005)	**0.003**
Full − SER	0.738 (0.696, 0.781)	0.421	− 0.007 (− 0.014, 0.0003)	0.060
Full − BCVA	0.741 (0.698, 0.784)	0.684	− 0.002 (− 0.006, 0.002)	0.363
Full − IOP	0.742 (0.699, 0.784)	0.851	− 0.001 (− 0.003, 0.001)	0.509
Age + ChT + Sex	0.735 (0.692, 0.778)	0.256	− 0.011 (− 0.020, − 0.002)	**0.021**
Age + ChT	0.721 (0.677, 0.765)	**0.025**	− 0.026 (− 0.040, − 0.013)	** < 0.001**
** ≥ 0.15 mm**^**b**^				
Full model 3	0.819 (0.774, 0.864)	Ref	Ref	
Full − Age	0.727 (0.661,0.792)	0.001	− 0.096 (− 0.134, − 0.058)	**0.001**
Full − ChT	0.787 (0.736, 0.837)	0.037	− 0.043 (− 0.070, − 0.017)	**0.001**
Full − AL	0.803 (0.756, 0.851)	0.132	− 0.025 (− 0.046, − 0.003)	**0.025**
Full − SEX	0.814 (0.769, 0.858)	0.489	− 0.016 (− 0.030, − 0.003)	**0.016**
Full − CVI	0.816 (0.771, 0.861)	0.545	− 0.007 (− 0.018, 0.005)	0.250
Full − BCVA	0.820 (0.776, 0.865)	0.710	− 0.003 (− 0.008, 0.002)	0.294
Full − IOP	0.819 (0.774, 0.864)	0.696	0.000 (− 0.001, 0.001)	0.915
Age + ChT + Sex	0.798 (0.750, 0.845)	0.109	− 0.035 (− 0.062, − 0.009)	**0.008**
Age + ChT	0.795 (0.748, 0.842)	0.082	− 0.044 (− 0.070, − 0.017)	**0.001**

The full model 1 included age, ChT, sex, AL, CVI, and BCVA

The full model 2 included age, ChT, sex, SER, BCVA, and IOP

The full model 3 included age, ChT, sex, AL, CVI, BCVA, and IOP

*AL* = axial length; *BCVA* = best-corrected visual acuity; *ChT =* choroidal thickness; *CI* = confidence interval; *CVI* = choroidal vascularity index; *IDI* = integrated discrimination improvement; *IOP* = intraocular pressure; *Ref* = reference; *SER* = spherical equivalent refraction

^a^ Delong test

^b^ Cutoffs of rapid axial elongation

Given the critical contribution of these key variables, two simplified models were developed. A model incorporating ChT, age, and sex achieved good discrimination (AUC = 0.794, 95% CI: 0.750 to 0.838), which was comparable to that of the full model (DeLong test, *P* = 0.084). Compared with the full model, it yielded a mild compromise in IDI of − 0.028 (95% CI:  − 0.048 to − 0.008; *P* < 0.05; Table 3). Likewise, a bivariate model containing only ChT and age performed comparably to the full model (AUC = 0.793, 95% CI: 0.749 to 0.836; DeLong test, *P* = 0.067), but with a modest decline in IDI (− 0.033, 95% CI:  − 0.054 to − 0.012; *P* < 0.05). Notably, sensitivity analyses using alternative axial elongation definitions (0.05 mm and 0.15 mm) confirmed the robustness of these findings, with consistent patterns of predictor importance and stable model performance (Table 3).

**Fig. 2:**
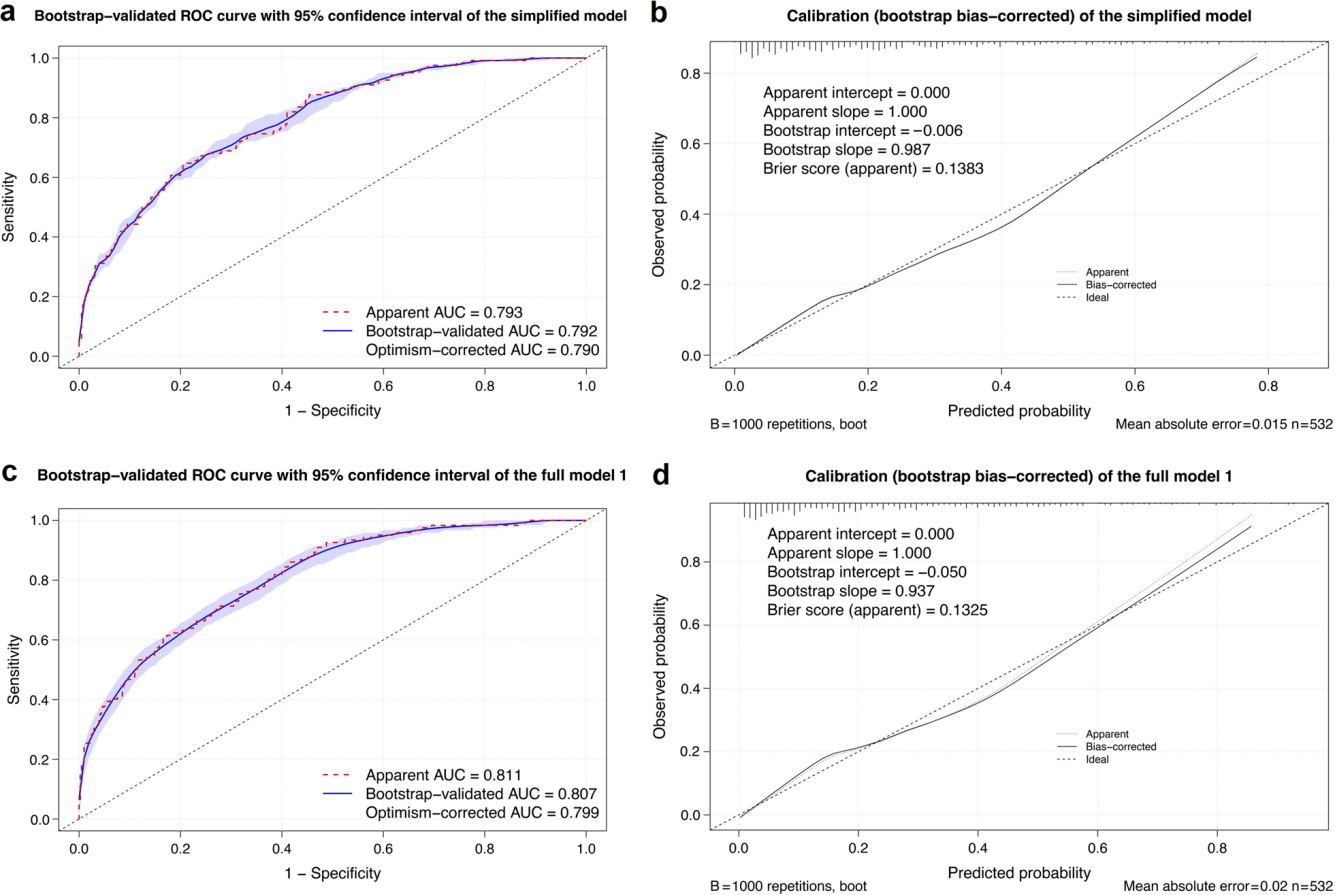
Development and internal validation of the model for predicting rapid axial elongation (≥ 0.1 mm). **a**, **b** ROC curve and calibration curves of the simplified model (age + choroidal thickness). **c**, **d** ROC curve and calibration curves of the full model 1. AUC, area under the curve; ROC, receiver operating characteristic

**Fig. 3:**
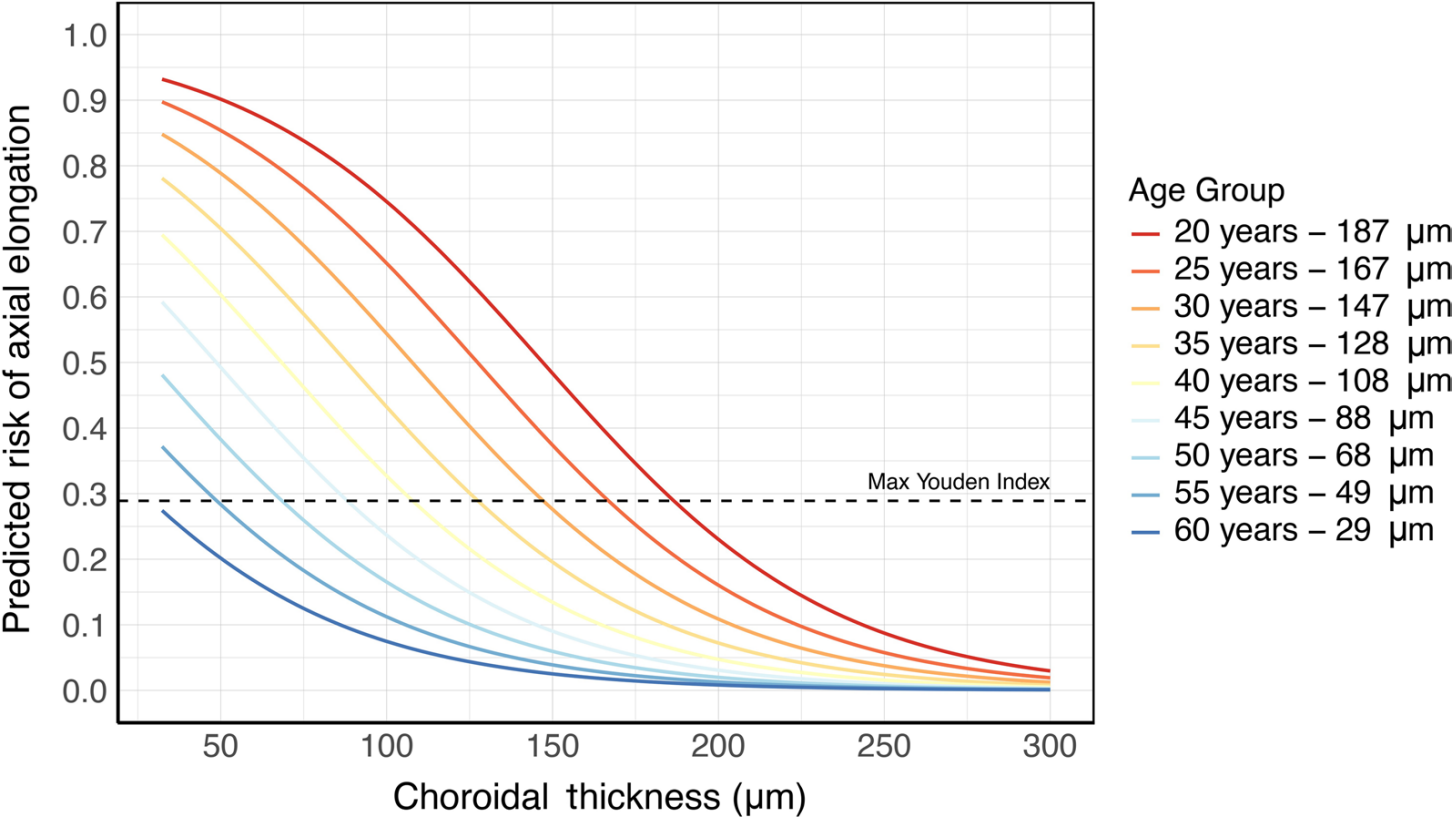
Risk score for predicting axial elongation based on age and choroidal thickness. The legend presents the key choroidal thickness cutoffs for each age group determined by the maximum Youden index

Internal validation using 1000 bootstrap resamples confirmed the model stability. The full model yielded an optimism-corrected AUC of 0.799 (apparent AUC = 0.811), while the simplified model (age + ChT) showed an optimism-corrected AUC of 0.790 (apparent AUC = 0.793). The negligible optimism bias (0.003) in the simplified model indicates a low risk of overfitting (Fig. 2a, c). Calibration plots (Fig. 2b, d) showed excellent agreement between predicted and observed probabilities of rapid axial elongation in both the simplified model (age + ChT) and the full model 1, indicating superior calibration performance. Finally, a probability distribution plot was generated (Fig. 3) to elucidate the dynamic relationship between risk and the key predictors (age + ChT). Visualization showed a progressive, age-dependent decline in the optimal ChT cut-off, decreasing from 187 µm at age 20 to 29 µm at age 60. Within this model, the predicted risk of rapid axial elongation increased as ChT decreased for a given age.

### Relationship of ChT and age with annual axial elongation rate

**Fig. 4:**
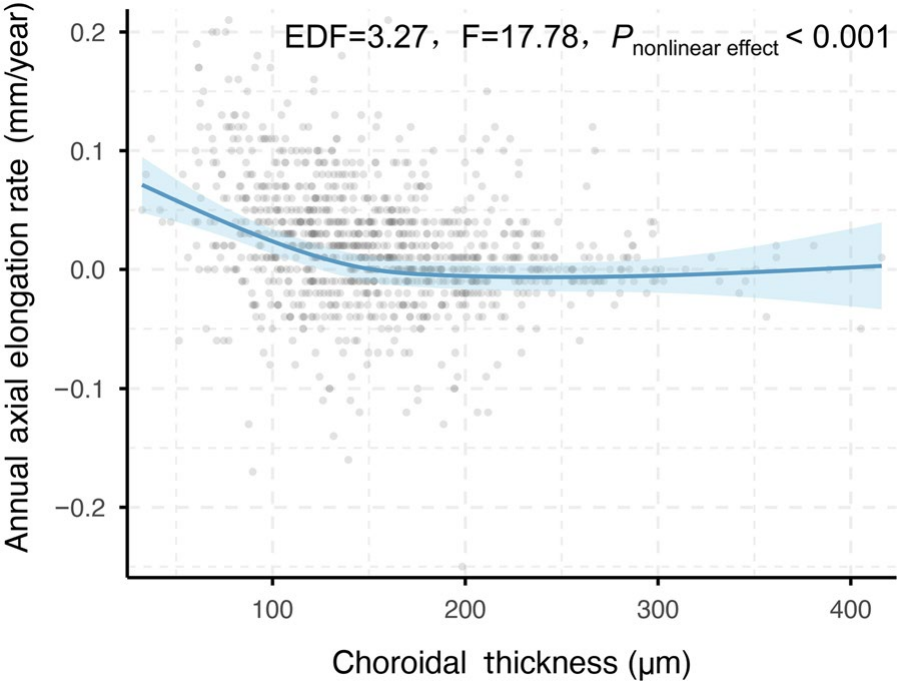
Association between baseline choroidal thickness and annual axial elongation rate.

To further characterize the relationship between key predictors and axial elongation, LOWESS smoothing was applied to visualize the trends. The analysis showed an approximately linear correlation between age and annual axial elongation rate, while ChT exhibited a distinct nonlinear pattern (Additional File 1 Figure S4). This finding was confirmed using a GAMM adjusted for age, sex, baseline AL, baseline refraction, visual acuity, and myopic maculopathy. The GAMM confirmed a significant nonlinear relationship between baseline ChT and the annual axial elongation rate (EDF = 3.27, F = 17.78, *P* < 0.001; Fig. 4). Specifically, a threshold effect was observed between baseline ChT and the annual axial elongation rate, with an inflection point near the Youden index‑derived cut-off of 138µm. Below this point was a thinner choroid associated with a higher annual axial elongation rate. This nonlinear relationship was consistently observed in younger and older subgroups (Additional File 1 Figure S5). Collectively, this threshold effect clarifies why ChT showed comparatively high discriminative power among the candidate predictors.

## Discussion

Identifying adults with high myopia who are at high risk of axial elongation is crucial for managing this condition, as further axial elongation could exponentially increase their lifetime risk of vision-threatening complications. In this study, we identified a set of candidate predictors, including age, sex, AL, BCVA, ChT, and CVI. Among these, ChT emerged as the single best predictor for axial elongation in adults with high myopia. Furthermore, a combination of ChT and age yielded the simplest predictive model while maintaining considerable predictive performance and calibration. The observed threshold effect between baseline ChT and the annual axial elongation rate further underscores the unique value of ChT in the risk stratification model. These findings demonstrated the clinical applicability of potential risk factors, thereby facilitating risk stratification and personalized management of axial elongation in adults with high myopia.

### Interpretation of predictive factors

Beyond corroborating potential risk factors, this study further refines our insight into these factors by identifying the optimal predictive factors. Consistent with previous studies [3, 4, 6, 9–11], younger age and female sex were independently associated with an increased risk of axial elongation, thereby highlighting their importance as non-modifiable demographic factors. Regarding ocular parameters, AL and SER were not retained concurrently in the models across all axial elongation definitions, suggesting limited explanatory power for axial elongation risk. Specifically, SER was only a predictor in the 0.05 mm threshold model, while AL was included in the higher-threshold models (0.10 and 0.15 mm), indicating that AL, as a key determinant of globe architecture, may be the critical factor driving axial elongation risk. Nevertheless, AL alone cannot fully account for the anatomical variability of the eyeball [17, 18]. This point clarifies the inclusion of ChT through stepwise regression, which conveys additional information about potential complications [19] (e.g., MMD) beyond the aforementioned risk factors [17]. Notably, age, sex, BCVA, and ChT were consistently retained as candidate predictors across models employing different axial elongation cut-offs. Among these factors, BCVA made a relatively smaller contribution, whereas age, sex, and ChT demonstrated greater importance, highlighting their potential clinical relevance.

While stepwise regression facilitated variable selection, notable differences were observed in the predictive performance of the selected individual factors. ChT demonstrated the highest discriminative ability, yielding an AUC of 0.728 when used alone, whereas the AUC values of other individual predictors ranged from 0.510 to 0.666. Furthermore, excluding ChT from the full multivariable model resulted in a decline in overall predictive accuracy, with the AUC decreasing to a level only marginally higher than that achieved by ChT alone. ChT exhibited the largest effect size among all variables in univariable analysis; however, age emerged as the stronger predictor in the multivariable model. These results indicate that ChT and age are the optimal predictors for axial elongation in adults with high myopia.

Evidence from animal models and children suggests that reduced ChT and impaired choroidal perfusion could be a risk factor for myopia development [20–26]. The present study revealed a nonlinear relationship between ChT and the rate of axial elongation: a pronounced increase in axial elongation rate occurred primarily when ChT fell below a certain threshold. Notably, this threshold closely approximated the optimal cut-off derived from the ROC curve analysis, reinforcing its use for clinical risk stratification. Finally, combining ChT with age further improved the model's predictive performance to a level that is comparable to that of the full model. Collectively, these findings support the use of age-specific ChT thresholds to guide clinical monitoring.

### Clinical implication of predictive models

The prediction model developed in this study occupies a distinct niche as a simple, transparent, and highly interpretable tool for assessing the risk of axial elongation. A key strength lies in its reliance on parameters routinely obtained during standard ophthalmic examinations. For example, ChT is easily measurable in clinical practice, and its automated segmentation is now commercially available on OCT platforms [17, 21, 27–29]. Despite its moderate discriminative capacity, the model’s excellent calibration and straightforward clinical applicability facilitate its integration into daily practice and enhance patient communication, overcoming a major barrier that has historically hindered the implementation of more complex predictive algorithms.

The prevalence of significant axial elongation among adults with high myopia varies considerably [2–5]. This variability underscores the influence of population-specific characteristics on disease progression. In the present cohort, the risk threshold optimized using the maximum Youden index corresponded to approximately 28.9%. This threshold appears conservative, but is clinically justified given the already elevated baseline ALs in this population. Therefore, proactive identification of individuals exceeding this threshold is critical to initiating timely and targeted interventions to forestall further progression.

Interventions to control progressive axial elongation in adults with high myopia may include specialized optical lenses, low-dose atropine, or posterior scleral reinforcement, although current recommendations are primarily based on expert consensus and extrapolations from pediatric myopia research [7, 30]. Nevertheless, early, risk-guided intervention may alter the disease trajectory, potentially slowing axial elongation and mitigating the lifetime risk of vision-threatening complications. Specifically, this predictive model facilitates a risk-stratified clinical management framework. For individuals categorized as high-risk (those with baseline ChT below the age-specific threshold), an intensified monitoring protocol is needed to facilitate early detection of rapid axial elongation and the potential development of vision-threatening complications. Therefore, clinicians should consider prioritizing proactive interventions for this subgroup, including specialized optical designs or surgical consultation for posterior scleral reinforcement, based on a personalized risk–benefit assessment. Conversely, for low-risk individuals, the model supports extended monitoring intervals, optimizing healthcare resource allocation. In addition, the individualized risk score serves as an objective metric to improve patient adherence to follow-up and lifestyle modifications by clarifying personal risk profiles. Beyond individualized care, this model offers broader utility, as it may inform routine diagnostic and therapeutic decisions, aid in preoperative risk stratification for refractive surgery candidates, and facilitate the enrichment of high-risk cohorts in future randomized controlled trials.

## Strengths and limitations

A principal strength of the current study is its prospective design, which facilitated the systematic collection of key variables under standardized protocols, including ChT measurement. Nevertheless, several limitations should be acknowledged. First, most participants with high myopia exhibited relatively healthy fundi and were in the early stages of myopic maculopathy. Consequently, the generalizability of our model to patients with advanced myopic maculopathy remains uncertain. However, this early-stage population is precisely where proactive risk stratification and intervention are most clinically actionable and yield the greatest benefits. Second, the analysis incorporated only a limited set of demographic factors and ocular parameters. Future investigations could integrate additional factors, such as genetic variations, to enhance the model’s predictive performance. Third, although internal validation was conducted, future studies should implement rigorous internal and external validation in independent cohorts to consolidate the reliability and applicability of our findings. Lastly, while our model effectively predicts axial elongation, future longitudinal studies with extended follow-up are required to confirm whether managing patients based on these risk profiles yields tangible benefits in preventing sight-threatening complications.

## Conclusions

This prospective cohort study validates a simple, transparent, and highly interpretable predictive model for progressive axial elongation in adults with high myopia. The findings underscore the pivotal roles of age and ChT in the personalized management of this population, laying a foundation for future research on risk prediction and real-world clinical application. Furthermore, these results highlight the need for future studies to investigate the role of choroidal architecture in the progression of high myopia. These efforts would provide theoretical insights and empirical support for proactive, choroid-centered strategies in myopia management.

## Supplementary Information

Additional file1 (DOCX 1294 KB)

## Data Availability

The data used and analyzed in this study are available from the corresponding author upon reasonable request.

## Abbreviations

AL: Axial length
BCVA: Best-corrected visual acuity
ChT: Choroidal thickness
SER: Spherical equivalent refraction
IOP: Intraocular pressure
MMD: Myopic macular degeneration
GAMM: Generalized additive mixed model
LOWESS: Locally weighted scatterplot smoothing
LA: Luminal area
IDI: Integrated discrimination improvement
SA: Stromal area
