# Supplementary material for: Prediction of axial elongation in adults with high myopia: the Wenzhou High Myopia Cohort Study
Source: Eye Vis (Lond). 2026 Jun 22;13:26. doi: 10.1186/s40662-026-00496-y (PMC13285501; doi:10.1186/s40662-026-00496-y)

**Figure S1.** Illustration of choroidal architecture analysis. **a** Structural optical coherence tomography (OCT) image. Scans along horizontal (yellow line) meridians was analyzed. **b** Original B-scan image. Scale: 3 mm (depth) × 12 mm (width). **c** Automatic segmentation of upper and lower choroidal boundary. **d** Binary images were generated using Niblack’s autolocal thresholding to segment the choroidal vascular luminal (LA) and stromal (SA) areas. Pixel-to-micron conversion was then applied using the image's pixel dimensions and B-scan parameters (adjusted for ocular magnification). **e** Overlays of binarized choroidal areas on original image. The 6-mm wide submacular area between the white lines centered on the fovea was used for analysis.


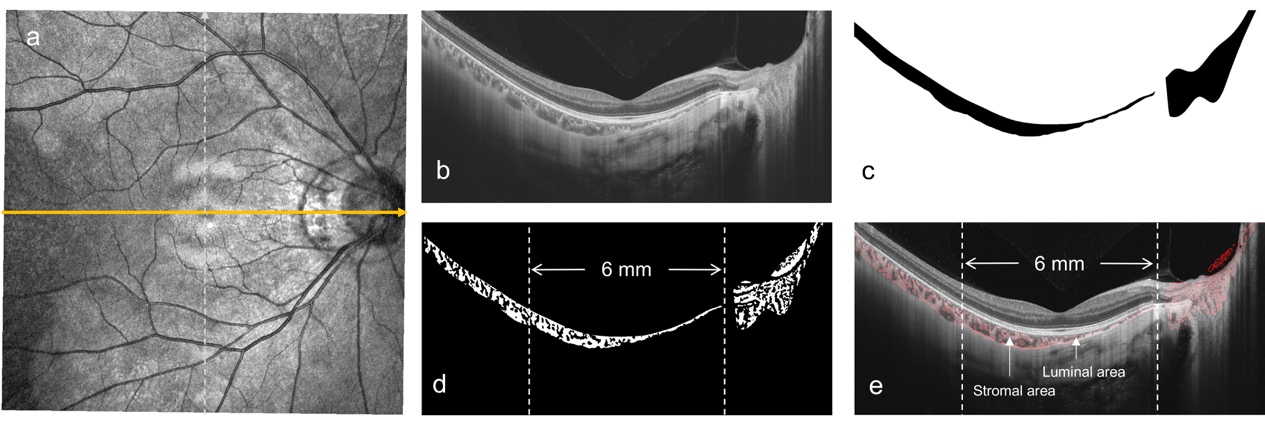


**Figure S2.** Receiver-operating characteristics curves for predicting rapid axial elongation (≥ 0.05 mm, ≥ 0.1 mm and ≥ 0.15 mm): (**a**) ≥ 0.05 mm (217 events among 532 eyes reaching threshold), (**b**) ≥ 0.1 mm (122/532), and (**c**) ≥ 0.15 mm (73/532). Sample size of 532 eyes. AL, axial length; BCVA, best-corrected visual acuity; AUC, area under the curve; ChT, choroidal thickness; CI, confidence interval; CVI, choroidal vascularity index; IOP, intraocular pressure; SER, spherical equivalent refraction.


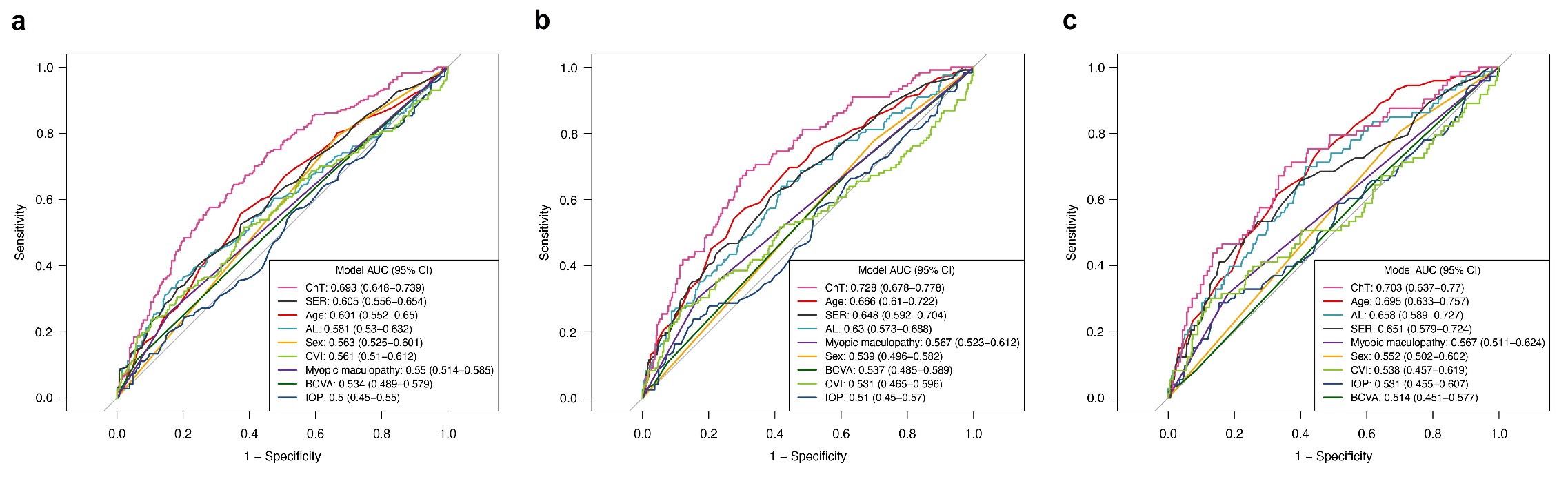


**Figure S3.** The importance of per feature of logistic model. Models are based on the following cut-offs: (**a**) ≥ 0.05 mm (217 events among 532 eyes reaching threshold), (**b**) ≥ 0.1 mm (122/532), and (**c**) ≥ 0.15 mm (73/532). Sample size of 532 eyes. BCVA, best-corrected visual acuity; IOP, intraocular pressure; SER, spherical equivalent refraction.


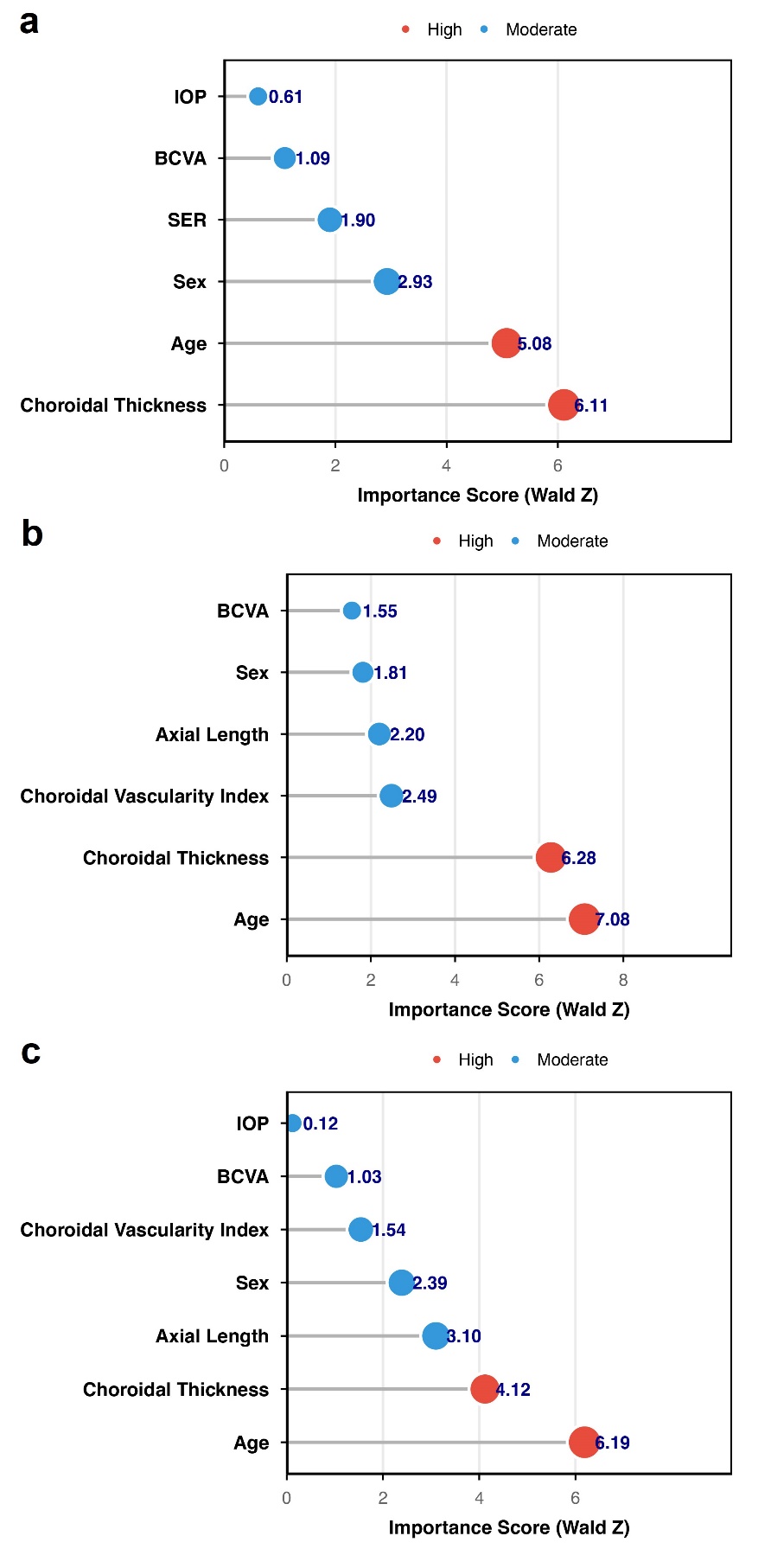


**Figure S4.** Scatter plots of baseline choroidal thickness (**a**) and age (**b**) with annual axial elongation rate. The curves were estimated with LOWESS plots with a smoothing value of 0.75.


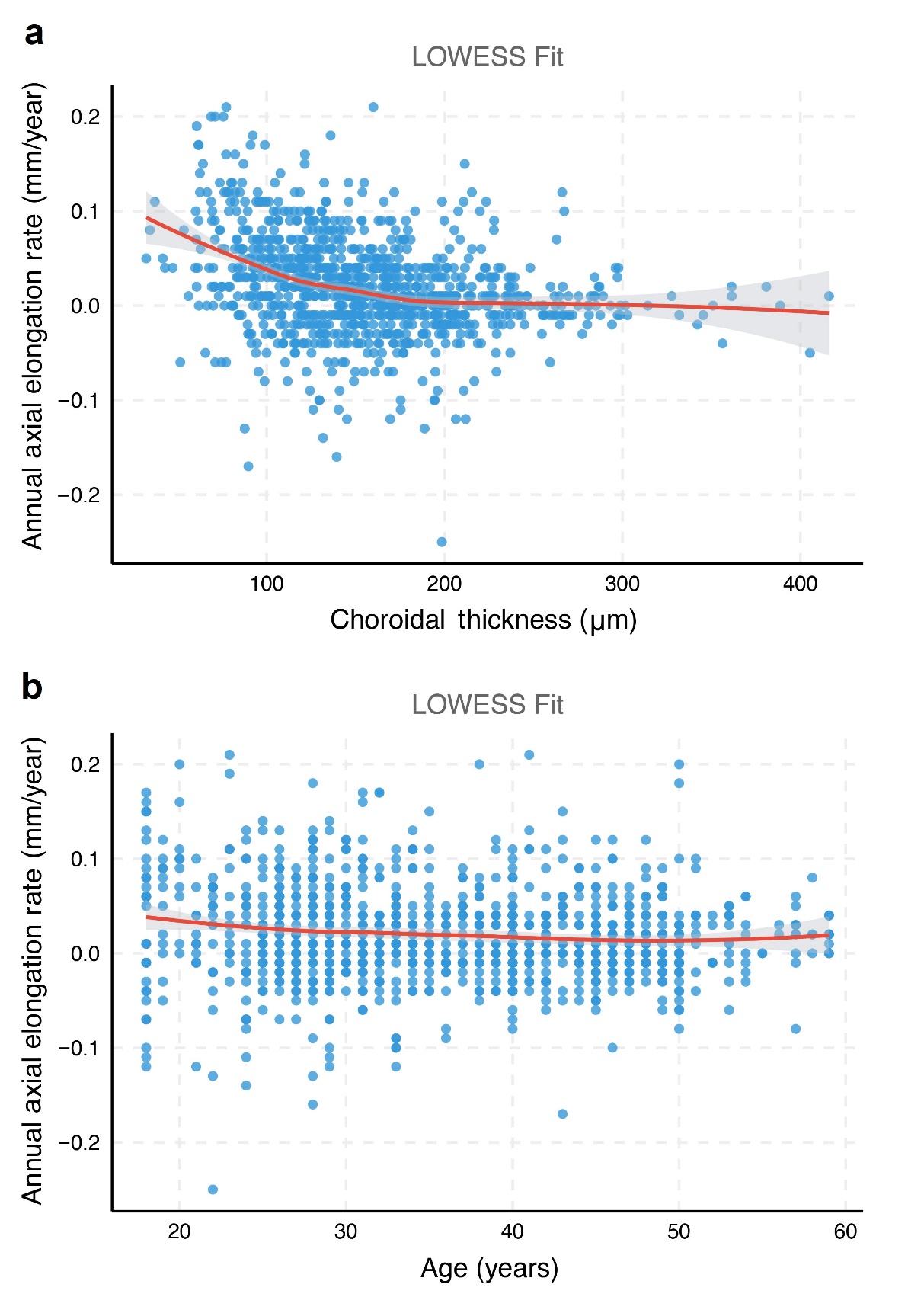


**Figure S5.** Association between baseline choroidal thickness with annual axial elongation rate in (**a**) younger (< 40 years) and (**b**) older subgroups (≥40 years). ChT, choroidal thickness


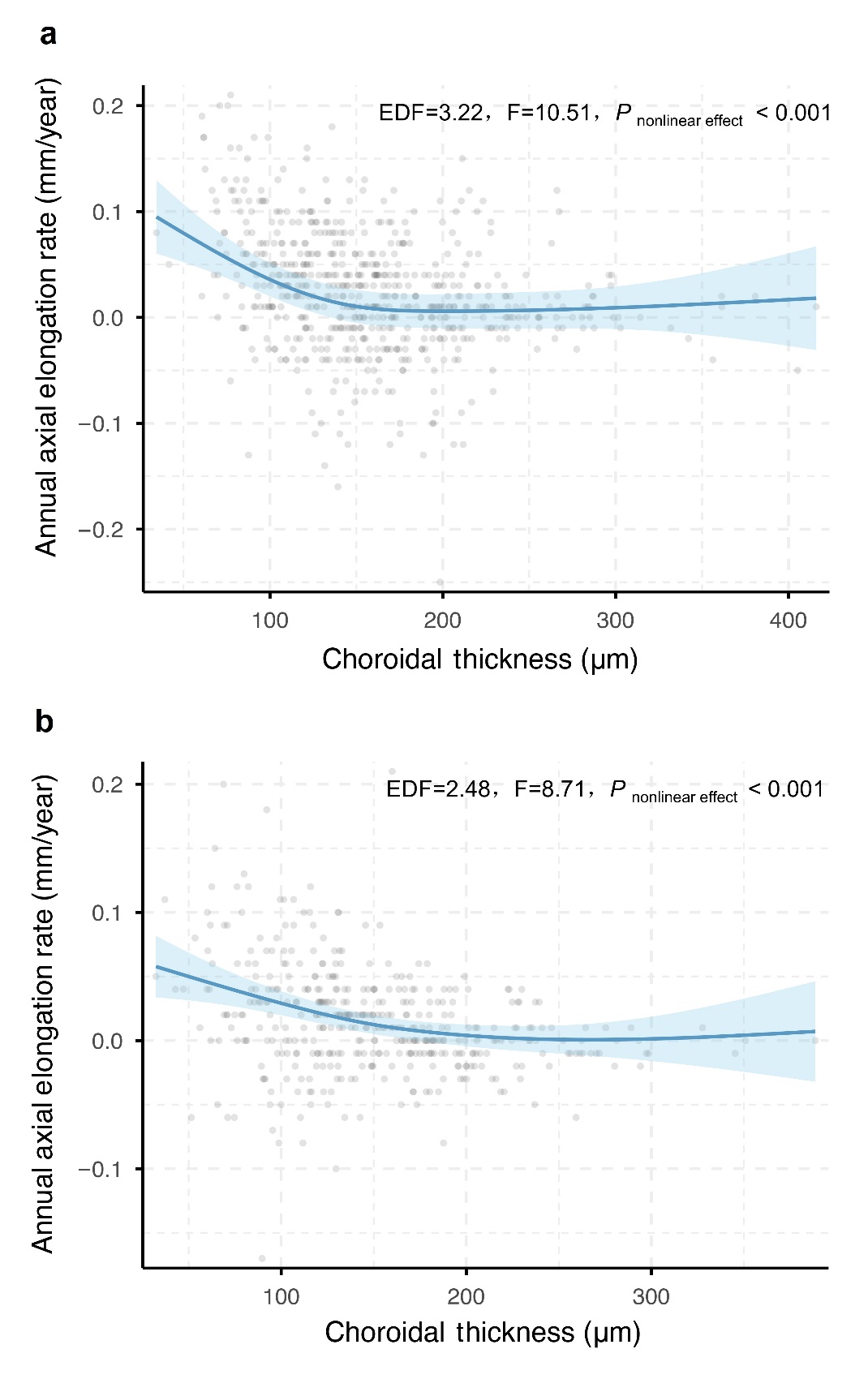

Supplement: Supplementary file 1 — Additional file1 (DOCX 1294 KB) [file 40662_2026_496_MOESM1_ESM.docx]
